# Prognostic nutritional index during hospitalization correlates with adverse outcomes in elderly patients with acute myocardial infarction: a single-center retrospective cohort study

**DOI:** 10.1007/s40520-024-02702-0

**Published:** 2024-03-05

**Authors:** Mingxuan Li, Jiasheng Cai, Kewei Jiang, Yanglei Li, Siqi Li, Qingyue Wang, Haibo Liu, Xinkai Qu, Chengqi Kong, Kailei Shi

**Affiliations:** 1 Department of Cardiology, Huadong Hospital Affiliated to Fudan University, Shanghai, China; 2 Department of Cardiology, Qingpu Branch of Zhongshan Hospital Affiliated to Fudan University, Shanghai, China; 3 Department of Respiratory Medicine, Huadong Hospital Affiliated to Fudan University, Shanghai, China

**Keywords:** Acute myocardial infarction, Prognostic nutritional index, Elderly, Prognosis

## Abstract

**Background and aims:**

Acute myocardial infarction (AMI) is one of the most prevalent illnesses endangering the elderly’s health. The predictive nutritional index (PNI) has been shown in several studies to be a good predictor of nutritional prognosis. In this study, we explored the correlation between PNI during hospitalization and the outcome of elderly AMI patients.

**Methods:**

Elderly AMI patients in the Cardiac Intensive Care Unit of Huadong Hospital from September 2017 to April 2020 were recruited for analysis. The clinical and laboratory examination data of subjects were retrieved. All enrolled patients were monitored following discharge. The primary clinical endpoints encompass major adverse cardiovascular events (MACEs) and Composite endpoint (MACEs and all-cause mortality). Survival analyses were conducted via the Kaplan–Meier and the log-rank analyses, and the Cox, proportional hazards model, was employed for hazard rate (HR) calculation.

**Results:**

307 subjects were recruited for analysis. The optimal PNI threshold is 40.923. Based on the Kaplan–Meier analysis, the elevated PNI group experienced better prognosis (*P* < 0.001). Cox analysis demonstrated that the PNI group was a stand-alone predictor for elderly AMI patient prognosis (HR = 1.674, 95% CI 1.076–2.604, *P* = 0.022). Subgroup analysis showed that the HR of the PNI group was the highest in the ST-segment elevation myocardial infarction (STEMI) subgroup (HR = 3.345, 95% CI 1.889–5.923, *P* = 0.05), but no discernible difference was observed in the non-ST-segment elevation myocardial infarction (NSTEMI) subgroup.

**Conclusion:**

Based on our analyses, the PNI during hospitalization can accurately predict the prognosis of elderly STEMI patients but not that of elderly NSTEMI patients.

## Introduction

Acute myocardial infarction (AMI) is a kind of myocardial ischemia and necrosis caused by a sharp reduction in coronary blood supply [1]. AMI, unlike other cardiovascular disorders, has an acute onset, high mortality, and poor prognosis. Although the mortality rate of patients with AMI has decreased with the advancement of coronary stent technology and with an improvement of the emergency percutaneous coronary intervention (PCI) process, it remains one of the primary illnesses impacting human health. The prevalence and death rate of AMI increase with age [2, 3]. In the United States, more than 60% of AMI occurs in the elderly. Compared with younger patients, elderly patients have a higher risk of arrhythmia, heart failure, and mechanical complications, as well as having a low cardiac reserve and a variety of underlying disorders. More than 60% of those who died from a myocardial infarction were above than 75 years [2]. Therefore, early detection of a poor prognosis of elderly individuals with AMI is essential.

The proportion of malnutrition in the elderly population is higher. When individuals have both cardiovascular disease and malnutrition, their death rate rises. The prognostic nutritional index (PNI) is a simple index reflecting human nutritional and immunity levels [4]. Many studies have shown that the PNI can predict the prognosis of tumors [5, 6], autoimmune diseases [7], chronic obstructive pulmonary disease (COPD) [8], acute heart failure [9], cardiomyopathy [10], and other diseases [11–13]. Nevertheless, there have been few and contentious studies on the relationship between PNI and AMI prognosis. Previous investigations revealed that the PNI can serve as a prognostic indicator of acute ST-segment elevation myocardial infarction (STEMI) [14, 15]. However, the link between non-ST-segment elevation myocardial infarction (NSTEMI) and the PNI remains undetermined. Alyoncuoğlu et al. indicated that PNI did not reflect the prognosis of NSTEMI patients. In another investigation, researchers discovered that PNI could successfully predict the probability of death [16–18]. In addition, most previous researchers have not conducted subgroup analyses based on age. Therefore, it is necessary to further clarify whether there is a correlation between PNI and elderly AMI patient prognosis.

Herein, we explored a possible association between PNI during hospitalization and undesirable prognosis among elderly AMI patients.

## Methods

### Research object

Patients with AMI hospitalized at the Cardiac Intensive Care Unit of Huadong Hospital (affiliated with Fudan University) from September 2017 to April 2020 were eligible for analysis. The following patients were included in our study: 1. In line with the 2018 global diagnostic criteria for AMI; 2. Age ≥ 60 years. Exclusion criteria included: (1) Patients whose serum albumin and/or lymphocyte counts were not measured; (2) Tumor patients; (3) Severe liver and kidney dysfunction; (4) In the acute infection period; (5) Refusing to participate in this study; (6) Patients were lost during follow-up. PNI calculation method: serum albumin (g/L) + 5 × lymphocyte count (10^9). The optimal PNI threshold was calculated via a receiver operating characteristic (ROC) curve. Based on the PNI threshold value, participants were then separated into two groups: elevated PNI and reduced PNI cohorts.

### Research methods

**Fig. 1:**
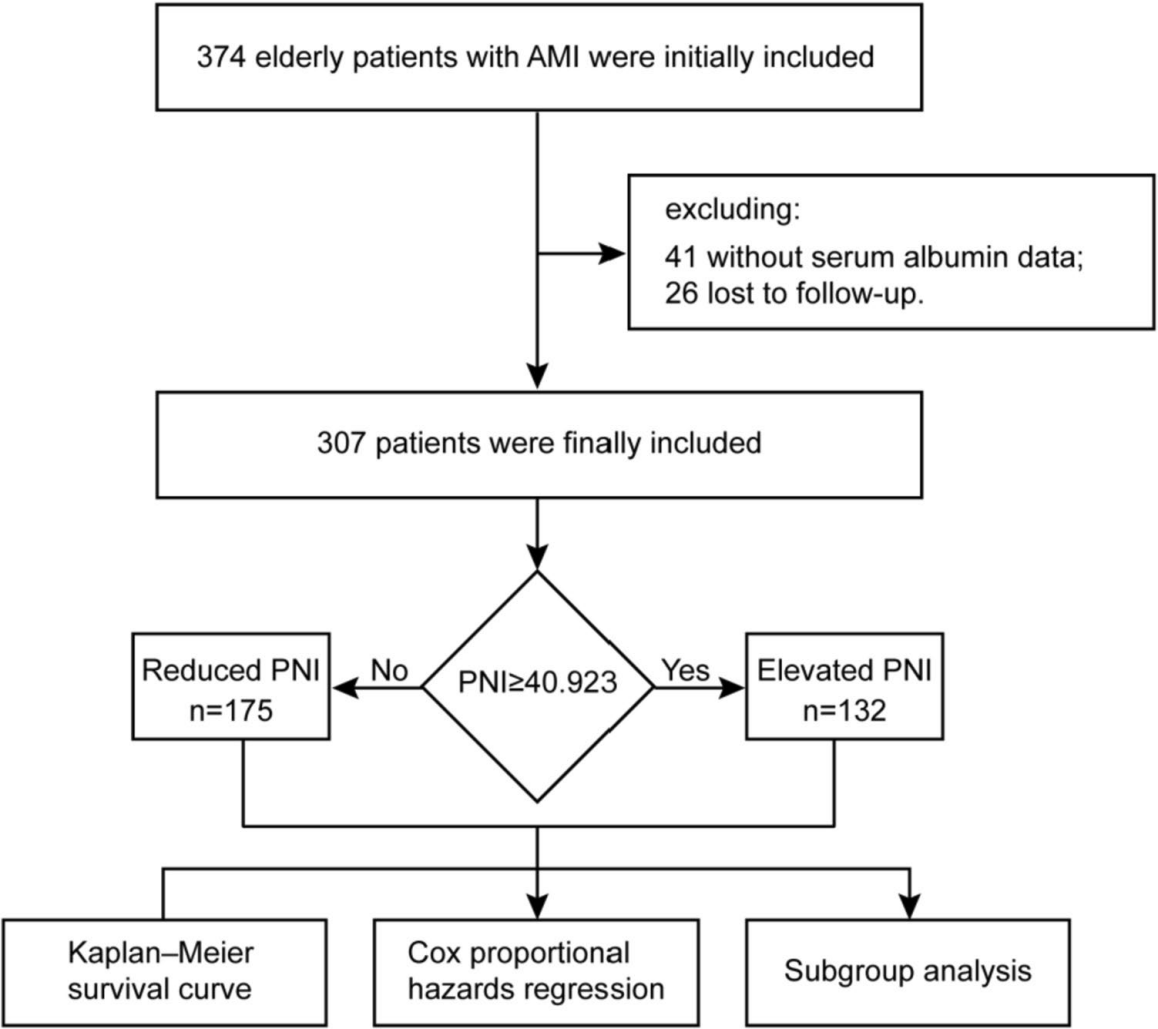
Study flow chart. *AMI* Acute myocardial infarction, *PNI* prognostic nutritional index

The cohort research was retrospective, involving a single-center. At the time of admission, the following clinical data about the patient was recorded: age, sex, height, weight, body mass index (BMI), history of hypertension and diabetes, history of smoking and alcohol use, type of myocardial infarction, and Killip classification [19]. The following indicators of the laboratory examination were also recorded: hemoglobin (HB), alanine aminotransferase (ALT), aspartate aminotransferase (AST), serum albumin (SA), urea nitrogen, serum creatinine (SCR), estimated glomerular filtration rate (eGFR), serum uric acid (sUA), total cholesterol (TC), triglyceride (TG), low-density lipoprotein (LDL), high-density lipoprotein (HDL), cardiac troponin T (cTnT), and creatine kinase myocardial band (CKMB). Clinical data were collected by two experienced cardiologists using the Huadong Hospital’s HIS system. The subjects were kept on an empty stomach for 8 h or more before blood collection, and 5 mL of elbow venous blood was collected by the nurse. After the serum was extracted, the automatic biochemical analyzer cobas® 8000 was used for analysis. The diagnostic criteria for hypertension included: clinic blood pressure (SBP ≥ 140 mmHg and/or DBP ≥ 90 mmHg) recorded thrice daily without using antihypertensive drugs; the patient had a history of hypertension. The diabetic diagnosis was based on the following: fasting blood glucose (BG) ≥ 7.0 mmol/L or 2 h postprandial BG ≥ 11.1 mmol/L. The CKD-EPI formula was used to calculate the eGFR [19]. Outpatient or telephone follow-up took place once every 6 months following discharge by one cardiovascular physician. The last follow-up time was in September 2021. This investigation received ethical approval from our institution, and informed consent from all subjects before the study. Our study design is displayed in Fig. 1.

### The primary clinical endpoints of the study

The primary clinical endpoints of this study encompass the following: (1) major adverse cardiovascular events (MACEs), comprising acute coronary syndrome, unplanned percutaneous coronary intervention (PCI), acute ischemic stroke, rehospitalization for acute heart failure, and malignant cardiac arrhythmias. Among these, ACS is defined as acute myocardial ischemia, including unstable angina, NSTEMI and STEMI. Malignant cardiac arrhythmias encompass sustained ventricular arrhythmias and high-degree atrioventricular conduction block. (2) Composite endpoint, consisting of MACEs and all-cause mortality. MACEs are based on the first/major diagnosis made by specialized medical institutions when the patient seeks treatment at the onset of the illness. Since some patients experience out-of-hospital mortality, making it challenging to ascertain the cause of death, we refrain from categorizing the causes of death. Instead, all deceased patients are classified under all-cause mortality.

### Statistical analysis

SPSS 25.0 and RStudio 4.0 were employed for all data analyses. Two-tailed Student *t*-test was employed for continuous variable assessment, and the *χ*^2^ test for dichotomous variable assessment. The ROC curve was generated via the pROC package. The Kaplan–Meier survival curve and the Cox proportional hazards regression model were utilized for survival analyses, and the Kaplan–Meier survival curve was generated using survminer package [20]. *P* < 0.05 was regarded as significant.

## Results

### Baseline information and ROC curve

**Fig. 2:**
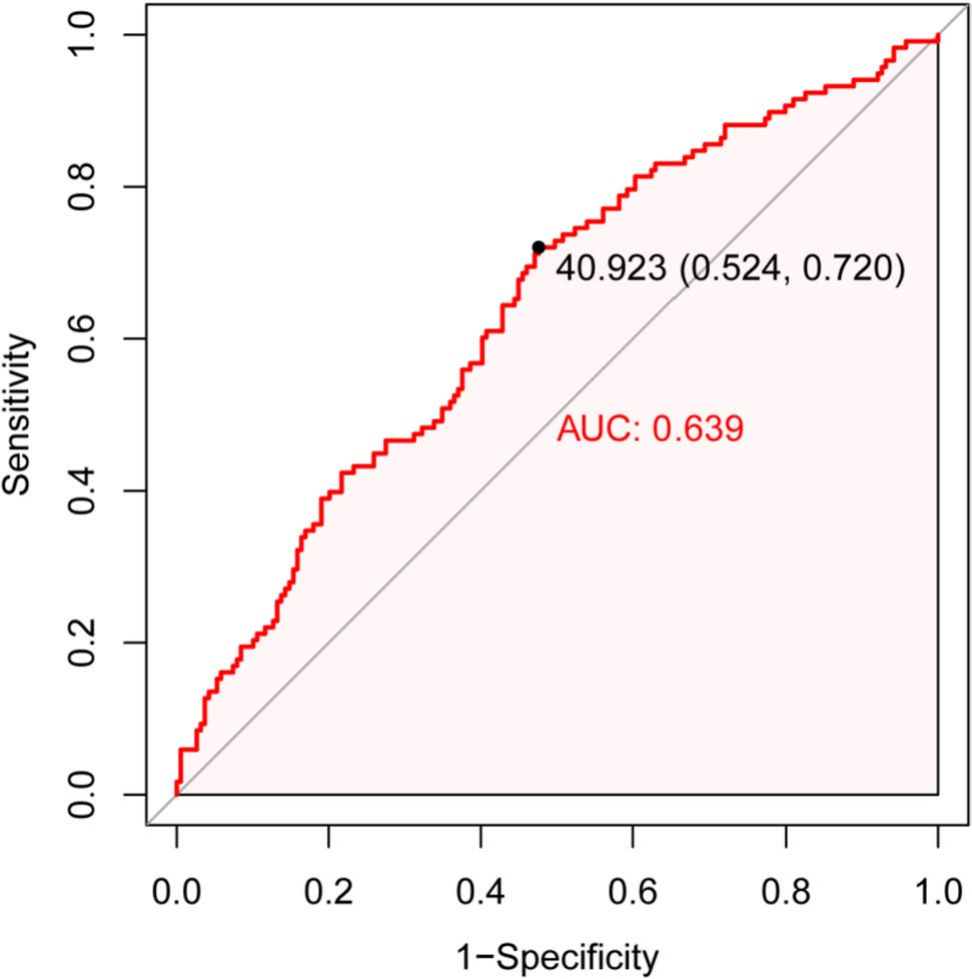
Receiver operating characteristic curve of PNI. *PNI* prognostic nutritional index, *AUC* area under the curve

This study initially included 374 elderly patients with AMI. After excluding 41 without serum albumin data and 26 lost to follow-up, 307 individuals were finally included. The mean follow-up duration was 27.3 ± 14.9 months, and the study included 208 (67.8%) males and 99 (32.2%) females, averaging 75.28 ± 9.44 years of age (Table 1). According to the calculated PNI value, the ROC curve was generated. The results are presented in Fig. 2. The area under the curve was 0.639, the sensitivity and specificity were 72.0% and 52.4%, respectively, and the optimal threshold was 40.923. The patients were next separated into elevated and reduced PNI cohorts, based on the optimal threshold value. Among the 132 people in the elevated PNI cohort, 89 (67.4%) were males and 43 (32.6%), were females, averaging 71.94 ± 8.83 years of age; meanwhile, among the 175 patients in the reduced PNI cohort, 119 (68.0%) were males and 56 (32.0%) were females, averaging 77.79 ± 9.12 years of age. The results revealed significant differences in age, height, weight, BMI, history of smoking, Killip classification, lymphocyte count, HB, TC, TG, LDL, albumin, SCR, urea nitrogen, eGFR, CTNT, and PNI between the two cohorts (*P* < 0.05). The elevated PNI cohort age was drastically reduced, compared to the reduced PNI cohort, while the height, weight and BMI were elevated, relative to the reduced PNI cohort (Table 1).

**Table 1:** Baseline profiles of study population at admission

Characteristics	No. of patients (*n* = 307)	Elevated PNI (*n* = 132)	Reduced PNI (*n* = 175)	*P* value
Age (years), mean ± SD	75.28 ± 9.44	71.94 ± 8.83	77.79 ± 9.12	< 0.001
Height (cm), mean ± SD	165.96 ± 7.97	167.13 ± 8.06	165.08 ± 7.81	0.025
Weight (kg), mean ± SD	67.00 ± 11.69	69.40 ± 11.22	65.19 ± 11.73	0.002
BMI (kg/m^2^), mean ± SD	24.25 ± 3.47	24.75 ± 2.87	23.88 ± 3.82	0.03
Sex, *n* (%)				1
Male	208 (67.8)	89 (67.4)	119 (68.0)	
Female	99 (32.2)	43 (32.6)	56 (32.0)	
Hypertension, *n* (%)				0.721
Yes	223 (72.6)	94 (71.2)	129 (73.7)	
No	84 (27.4)	38 (28.8)	46 (26.3)	
Diabetes, *n* (%)				0.589
Yes	104 (33.9)	42 (31.8)	62 (35.4)	
No	203 (66.1)	90 (31.8)	113 (64.6)	
Smoking, *n* (%)				0.007
Yes	92 (30.0)	52 (39.4)	40 (22.9)	
No	188 (61.2)	70 (53.0)	118 (67.4)	
Stop	27 (8.8)	10 (7.6)	17 (9.7)	
Drinking, *n* (%)				0.459
Yes	47 (15.3)	24 (18.2)	23 (13.1)	
No	257 (83.7)	107 (81.1)	150 (85.7)	
Stop	3 (1.0)	1 (0.8)	2 (1.1)	
Killip classification, *n* (%)				0.005
I	217 (70.7)	106 (80.3)	111 (63.4)	
II	49 (16.0)	16 (12.1)	33 (18.9)	
III	12 (3.9)	1 (0.8)	11 (6.3)	
IV	29 (9.4)	9 (6.8)	20 (11.4)	
MI type, *n* (%)				0.291
STEMI	151 (49.2)	70 (53.0)	81 (46.3)	
NSTEMI	156 (50.8)	62 (47.0)	94 (53.7)	
sWBC (10^9^), mean ± SD	9.52 ± 3.47	9.46 ± 2.98	9.56 ± 3.81	0.79
LYM (10^9^), mean ± SD	1.38 ± 0.65	1.64 ± 0.74	1.18 ± 0.49	< 0.001
HB (g/L), mean ± SD	125.47 ± 20.28	134.26 ± 16.65	118.84 ± 20.30	< 0.001
ALT (U/L), median (IQR)	27.80 [19.00, 44.00]	26.50 [19.00, 44.00]	28.10 [18.00, 43.50]	0.96
AST (U/L), median (IQR)	65.00 [33.00, 153.00]	61.00 [31.50, 155.50]	71.00 [35.00, 148.50]	0.652
TC (mmol/L), mean ± SD	4.13 ± 1.00	4.46 ± 0.89	3.88 ± 1.01	< 0.001
TG (mmol/L), median (IQR)	1.30 [1.00, 1.90]	1.60 [1.20, 2.00]	1.20 [0.90, 1.60]	< 0.001
HDL (mmol/L), median (IQR)	1.12 [0.96, 1.31]	1.15 [1.01, 1.32]	1.11 [0.93, 1.29]	0.104
LDL (mmol/L), median (IQR)	2.32 [1.85, 2.84]	2.57 [2.11, 3.16]	2.13 [1.65, 2.62]	< 0.001
Albumin (g/L), median (IQR)	39.00 [36.00, 41.00]	42.00 [41.00, 43.00]	36.00 [34.20, 38.00]	< 0.001
sCR (μmol/L), median (IQR)	85.40 [71.05, 114.50]	79.75 [68.80, 100.43]	89.20 [73.05, 124.35]	0.004
UN (mmol/L), median (IQR)	5.90 [4.80, 7.85]	5.50 [4.80, 6.70]	6.70 [4.90, 9.05]	< 0.001
eGFR (mL/min), median (IQR)	70.55 [49.42, 90.11]	78.69 [59.16, 93.66]	65.75 [44.26, 83.44]	< 0.001

Characteristics	No. of patients (*n* = 307)	Elevated PNI (*n* = 132)	Reduced PNI (*n* = 175)	*P* value
sUA (μmol/L), median (IQR)	349.80 [287.65, 423.50]	348.00 [291.00, 418.35]	351.00 [286.00, 427.00]	0.447
CKMB (ng/mL), median (IQR)	36.10 [11.25, 116.15]	34.35 [11.15, 117.50]	36.90 [12.30, 114.20]	0.766
CTNT (ng/mL), median (IQR)	1.40 [0.41, 4.04]	1.04 [0.33, 2.93]	1.76 [0.58, 4.74]	0.016
PNI, median (IQR)	40.10 [37.20, 42.93]	43.27 [42.08, 45.14]	37.74 [35.44, 39.21]	< 0.001

*BMI* body mass index, *MI* myocardial infarction, *STEMI* ST-segment elevation myocardial infarction, *NSTEMI* non-ST-segment elevation myocardial infarction, *WBC* white blood cell, *LYM* lymphocyte, *HB* Hemoglobin, *ALT* alanine aminotransferase, *AST* aspartate aminotransferase, *TC* total cholesterol, *TG* triglyceride, *HDL* high density lipoprotein, *LDL* low density lipoprotein, *sCR* serum creatinine, *eGFR* estimated glomerular filtration rate, *sUA* serum uric acid, *UN* urea nitrogen, *CKMB* creatine kinase myocardial band, *CTNT* cardiac troponin T, *PNI* prognostic nutritional index

### Kaplan–Meier survival curve

**Fig. 3:**
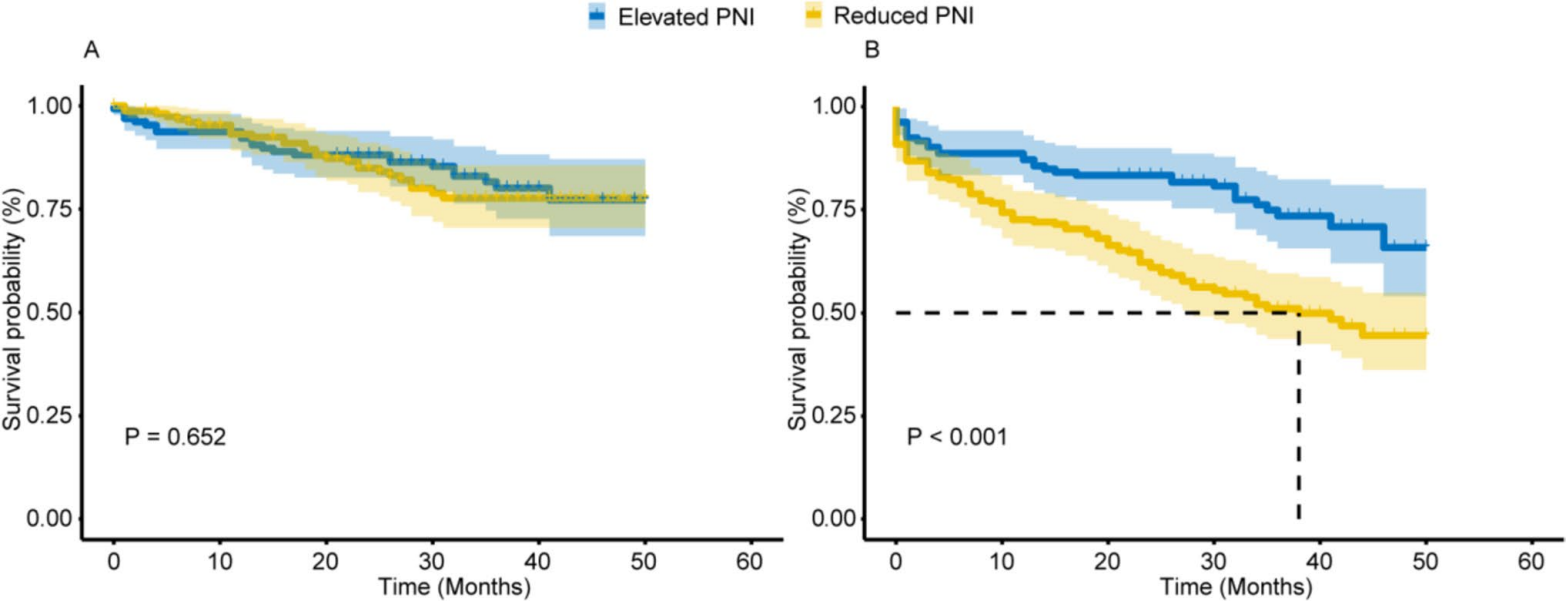
Kaplan–Meier survival curve. **A** MACEs serve as the endpoint; **B** the composite endpoint as the endpoint. The yellow line denotes subjects with reduced PNI, and the blue line denotes subjects with elevated PNI. *PNI* prognostic nutritional index, *MACEs* major adverse cardiovascular events. The composite endpoint includes MACEs and all-cause mortality

To elucidate the potential correlation between PNI and the primary clinical endpoints of elderly AMI patients, the Kaplan–Meier analysis was employed. As illustrated in Fig. 3. When major adverse cardiovascular events (MACEs) serve as the endpoint, there is no discernible disparity in prognosis between the two cohorts (Fig. 3A). However, when the composite endpoint of MACEs and all-cause mortality assumes primacy as the endpoint, the elevated PNI cohort exhibits a superior prognosis (*P*  <  0.001) (Fig. 3B).

### Cox regression analysis

To eliminate the influence of confounding factors upon the survival analysis, the representative variables (age, sex, height, weight, BMI, hypertension history, diabetes history, smoking and drinking histories, MI type, Killip classification, HB, ALT, eGFR, TC, TG, LDL, HDL, sUA, cTnT, PNI type) in the baseline data were first selected and included in the univariate Cox analysis. The associated data are presented in Table 2. Significant differences were observed in age, diabetes history, Killip classification, HB, ALT, eGFR, sUA, CTNT, and PNI groups (*P *< 0.05). The aforementioned variables were entered into multivariate analysis. The associated data are presented in Table 2. Age (HR = 1.034, 95% CI 1.008–1.059, *P* = 0.009), history of diabetes (HR = 1.732, 95% CI 1.186–2.527, *P* = 0.004), and PNI group (HR = 1.674, 95% CI 1.076–2.604, *P* = 0.022) were obviously significant and were stand-alone predictor of elderly AMI patient prognosis.

**Table 2:** Uni- and multivariate cox regression analyses of the main composite endpoints across the PNI

Characteristics	Univariate cox regression analysis			Multivariate cox regression analysis		
	Hazard ratio	95% CI	*P* value	Hazard ratio	95% CI	*P* value
Age	1.052	1.032–1.072	< 0.001	1.034	1.008–1.059	0.009
Sex	1.152	0.787–1.687	0.467	–	–	–
Height	0.994	0.972–1.017	0.621	–	–	–
Weight	1.006	0.990–1.022	0.485	–	–	–
BMI	1.042	0.987–1.101	0.139	–	–	–
Hypertension	1.462	0.946–2.260	0.087	–	–	–
Diabetes	1.775	1.232–2.557	0.002	1.732	1.186–2.527	0.004
Smoking	0.853	0.638–1.141	0.284	–	–	–
Drinking	0.635	0.376–1.074	0.091	–	–	–
MI type	1.139	0.793–1.634	0.482	–	–	–
Killip classification	1.351	1.151–1.586	< 0.001	1.093	0.908–1.315	0.349
Hemoglobin	0.982	0.974–0.991	< 0.001	0.997	0.987–1.008	0.605
ALT	1.001	1.000–1.001	< 0.001	1.000	1.000–1.001	0.457
eGFR	0.982	0.975–0.989	< 0.001	0.998	0.987–1.009	0.746
Total cholesterol	0.859	0.715–1.032	0.105	–	–	–
Triglyceride	0.848	0.699–1.029	0.096	–	–	–
LDL	0.794	0.630–1.001	0.051	–	–	–
HDL	1.133	0.647–1.985	0.662	–	–	–
sUA	1.003	1.001–1.005	< 0.001	1.002	1.000–1.004	0.091
cTNT	1.092	1.038–1.149	0.001	1.050	0.992–1.111	0.091
PNI type	2.300	1.538–3.441	< 0.001	1.674	1.076–2.604	0.022

*BMI* body mass index, *MI* myocardial infarction, *ALT* alanine aminotransferase, *HDL* high density lipoprotein, *LDL* low density lipoprotein, *eGFR* estimated glomerular filtration rate, *sUA* serum uric acid, *CKMB* creatine kinase myocardial band, *CTNT* cardiac troponin T, *PNI* prognostic nutritional index, *CI* confidence interval

### Subgroup analysis

**Fig. 4:**
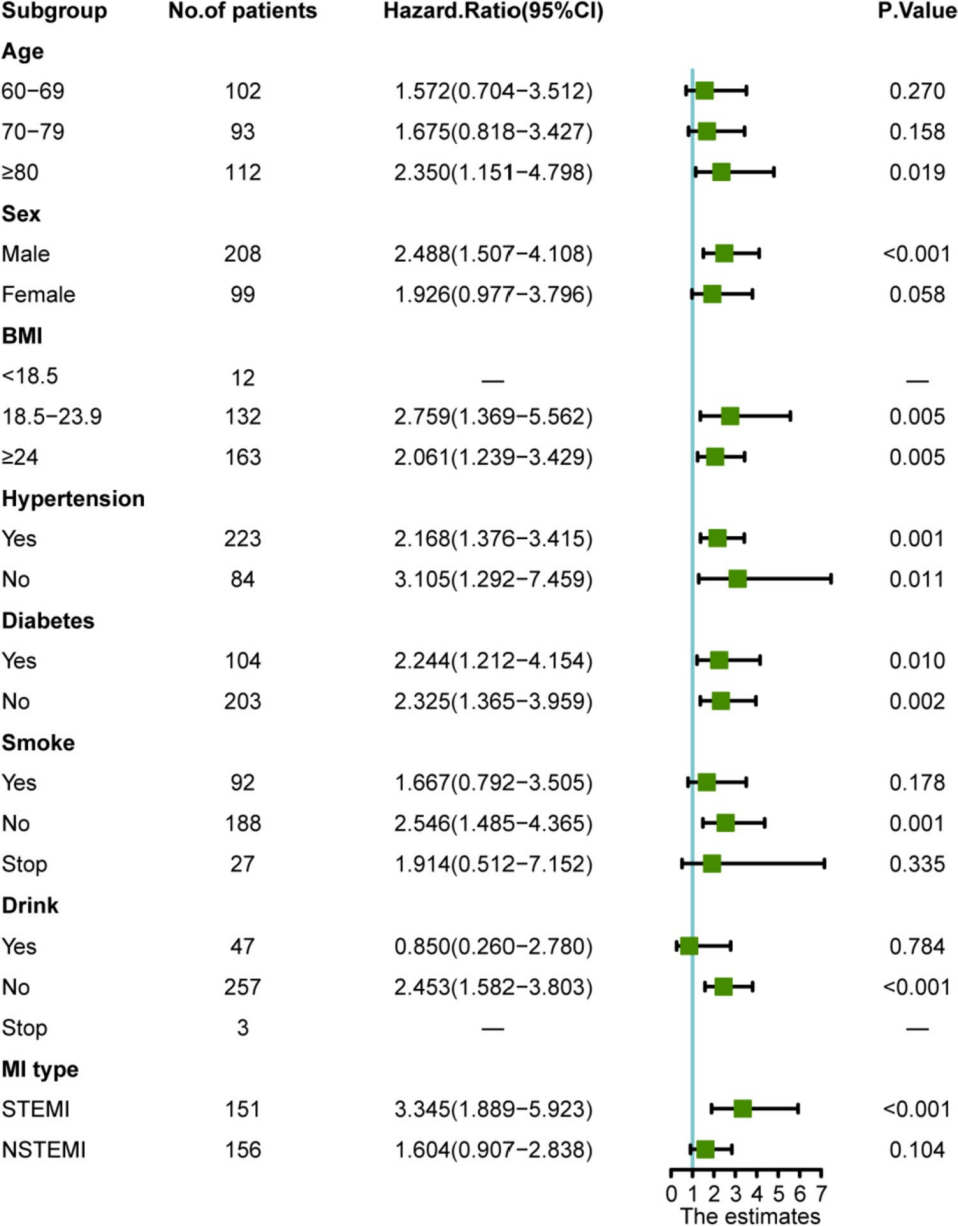
Subgroup analysis. *PNI* prognostic nutritional index, *BMI* body mass index, *MI* myocardial infarction, *STEMI* ST-segment elevation myocardial infarction, *NSETMI* non-ST-segment elevation myocardial infarction, *CI* confidence interval. The composite endpoint includes MACEs and all-cause mortality. *MACEs* major adverse cardiovascular events

Considering that the two groups of patients had differences concerning age, history of smoking, Killip classification, and other variables, to further explore the correlation between the PNI and prognosis in different populations, the Cox regression model was employed for the analysis of different subgroups. As illustrated in Fig. 4, the PNI group had the highest HR in STEMI subgroup (HR = 3.345, 95% CI 1.889–5.923, *P* = 0.05). No marked differences were observed in terms of those aged 60–79 years, females, smoking, smoking cessation, drinking, and NSTEMI patients (*P* > 0.05).

## Discussion

Overall, 307 elderly AMI patients were recruited for analysis. Firstly, the optimal PNI threshold via drawing a ROC diagram was 40.923. Following that, the study participants were separated into an elevated or reduced PNI cohort. We observed marked differences in patient age, height, weight, smoking history, and Killip classification between the two cohorts. The survival curve (as drawn by Kaplan and Meier) demonstrated that the elevated PNI cohort experienced enhanced prognosis. The Cox regression analysis depicted that the PNI group was a stand-alone predictor for in elderly AMI patient prognosis. In the subanalysis, we observed a highest risk of adverse outcomes in the subgroup of ST-segment elevation myocardial infarction (STEMI) individuals with the reduced PNI cohort.

In 1980, Buzby first proposed using PNI to evaluate outcome of patients undergoing gastrointestinal surgery [21]. He established a linear regression model of serum albumin, serum transferrin, triceps skinfold, and delayed hypersensitivity to reflect the nutritional status of patients. In 1984, Onodera modified the calculation of the PNI to retain only the albumin of the original model and added a new lymphocyte count to represent the immune and nutritional profile of patients [4]. In many studies on tumors, the ideal PNI threshold has been set at 45 [4, 22, 23]; that is to say, when the PNI is above 45, the prognosis of patients is better. In recent years, researchers have also explored the application of PNI in the prognosis of other diseases. Studies have found that the PNI also has a predictive effect on other tumors, namely, diffuse large B-cell lymphoma, breast cancer, and biliary tract cancer. In addition to tumors, researchers have expanded the application of the PNI to the prognosis of other diseases such as COPD, lung transplantation, dilated cardiomyopathy, and heart failure. In most studies, the ideal PNI threshold has been between 40 and 50 [24], such as 36.7 in patients with late-stage biliary tract cancer and 51 in patients with breast cancer [25, 26]. This study explored the optimal threshold of 40.923 in elderly patients with acute myocardial infarction. Combined with previous studies, we believe that different diseases have different pathogenesis and characteristics. Setting a uniform PNI cut-off value is not in line with clinical practice, and the critical value of each PNI should be set in different diseases.

Earlier investigations have explored the utility of PNI in prognosticating acute myocardial infarction (AMI) outcomes. In 2016, Basta et al. initially proposed an association between lower PNI levels and an unfavorable prognosis in elderly STEMI patients undergoing primary PCI [27]. Subsequently, both Chen et al. and Keskin et al. independently expanded their study populations to encompass all STEMI patients undergoing PCI, affirming PNI as an independent prognostic factor. Nevertheless, a consensus on whether PNI can independently predict outcomes in NSTEMI is currently lacking. Alyoncuoğlu et al. conducted a 1-year follow-up study involving 253 elderly NSTEMI patients, with major adverse cardiovascular events and mortality as the primary endpoint [18]. Their findings indicated that PNI did not reflect the prognosis of NSTEMI patients. Conversely, in two other clinical studies, researchers observed, through extended follow-ups of NSTEMI patients, that PNI could effectively predict the risk of mortality [16, 17]. In our study, we noted that PNI had no predictive value when MACEs served as the primary endpoint. However, when the endpoint included all-cause mortality, the cohort with elevated PNI exhibited a more favorable prognosis. In further subgroup analysis, we observed that PNI effectively reflected adverse outcomes in STEMI patients, whereas no such predictive effect was discerned in NSTEMI patients. We suppose that since all-cause mortality encompasses not only cardiovascular-related deaths but also those resulting from other conditions such as neoplasms, the predictive efficacy of PNI for the latter has been substantiated by numerous studies. Consequently, PNI may proficiently prognosticate comprehensive adverse outcomes in myocardial infarction patients. Additionally, we speculate that, compared to STEMI patients, the relatively milder severity of illness in NSTEMI patients may render the impact of unfavorable nutritional and immune states less pronounced, hence diminishing the predictive efficacy of PNI for NSTEMI patient outcomes. Besides, the heterogeneity in populations, diets, and cultures across diverse studies may impact the consistency of research findings. More extensive clinical research is warranted to delve deeper into these findings in the future.

## Conclusion

We found that the PNI during hospitalization can accurately predict the prognosis of elderly STEMI patients but not that of elderly NSTEMI patients.

## Limitations

This study still had certain deficiencies. First, it is a single-center investigation involving a modest patient population, and the influence of selection bias and mixed bias cannot be excluded. Second, due to the insufficient number of patients reaching study completion, the different outcomes of our trial cannot be analyzed; moreover, due to the limited research conditions, the patients were not followed up regarding the PNI after discharge; thus, the effect of PNI changes upon outcomes could not be determined.

## Data Availability

The data in this article is available, by contacting the corresponding author, upon reasonable request.
